# High glucose enhances progression of cholangiocarcinoma cells *via* STAT3 activation

**DOI:** 10.1038/srep18995

**Published:** 2016-01-08

**Authors:** Charupong Saengboonmee, Wunchana Seubwai, Chawalit Pairojkul, Sopit Wongkham

**Affiliations:** 1 Department of Biochemistry, Faculty of Medicine, Khon Kaen University, Khon Kaen, Thailand; 2 Department of Forensic Medicine, Faculty of Medicine, Khon Kaen University, Khon Kaen, Thailand; 3 Department of Pathology, Faculty of Medicine, Khon Kaen University, Khon Kaen, Thailand; 4 Liver Fluke and Cholangiocarcinoma Research Center, Faculty of Medicine, Khon Kaen University, Khon Kaen, Thailand

## Abstract

Epidemiological studies have indicated diabetes mellitus (DM) as a risk of cholangiocarcinoma (CCA), however, the effects and mechanisms of high glucose on progression of CCA remain unclear. This study reports for the first time of the enhancing effects of high glucose on aggressive phenotypes of CCA cells via STAT3 activation. CCA cells cultured in high glucose media exerted significantly higher rates of cell proliferation, adhesion, migration and invasion than those cultured in normal glucose. The phosphokinase array revealed STAT3 as the dominant signal activated in response to high glucose. Increased nuclear STAT3, p-STAT3 and its downstream target proteins, cyclin D1, vimentin and MMP2, were shown to be underling mechanisms of high glucose stimulation. The link of high glucose and STAT3 activation was confirmed in tumor tissues from CCA patients with DM that exhibited higher STAT3 activation than those without DM. Moreover, the levels of STAT3 activation were correlated with the levels of blood glucose. Finally, decreasing the level of glucose or using a STAT3 inhibitor could reduce the effects of high glucose. These findings suggest that controlling blood glucose or using a STAT3 inhibitor as an alternative approach may improve the therapeutic outcome of CCA patients with DM.

Diabetes Mellitus (DM), a disease characterized by high blood glucose, is globally increasing in both industrialized and developing countries^1^,^2^. Apart from its mild to serious complications, DM also increases risk for other non-communicable diseases including cancers. Not only an association with cancer risk, DM also promotes the progression of tumors resulting in a worse prognosis of patients in many types of cancer^3^,^4^.

Cholangiocarcinoma (CCA), is a malignancy that arises from the bile duct epithelia. Its incidence is considered low in the western countries but relatively high in Southeast Asia^5^. The highest incidence of CCA has been reported in the Northeast of Thailand where the infection of the liver fluke, *Opisthorchis viverrini* was shown to be a major risk factor^6^,^7^. Less than 1% of the *Opisthorchis viverrini*-infected individuals, however, develop CCA^8^,^9^, indicating that other individual risk factors may play a crucial role in CCA development.

Epidemiological studies have clearly demonstrated DM as one of the risk factors of CCA in the western countries^10^,^11^. In the current literature there is no direct study indicating the association of DM and CCA in Thai patients. The geographical mortality surveys in Thailand, however, have shown the overlay of geographical mortality of DM and CCA in northeast Thailand^12^. The possible risks and promoting factors of DM on CCA is currently reviewed^9^. Although, the effects of DM on progression and poor prognosis of cancer patients were repeatedly reported, the mechanisms by which DM or high blood glucose affects CCA remains unclear^13^. The understanding of interaction between the high glucose condition and CCA may unveil an alternative approach to improve the therapeutic outcome of CCA patients.

In the present study, it is demonstrated for the first time, the enhancing effect of high glucose upon the aggressive phenotypes of CCA cell lines, namely growth, adhesion, migration and invasion. The molecular pathway linking high glucose conditions to the aggressive properties of CCA was shown to be via STAT3 activation. The tie between high glucose and STAT3 activation was confirmed in CCA patients as nuclear p-STAT3 activation was significantly enhanced in tumor tissues of CCA patients with DM. These findings suggest the implication of controlling blood glucose or using a STAT3 inhibitor as a strategy for treatment of CCA patients with DM.

## Results

To test the effects of high glucose on the metastatic potential of CCA cells, two highly metastatic CCA cell lines, KKU-213 and KKU-214 were cultured in media containing normal glucose (N; 5.56 mM) or high glucose (H; 25 mM). Cells cultured in normal glucose media were designated as NG cells and those cultured in high glucose media were designated as HG cells.

### High glucose promotes CCA cells proliferation

**Figure 1:**
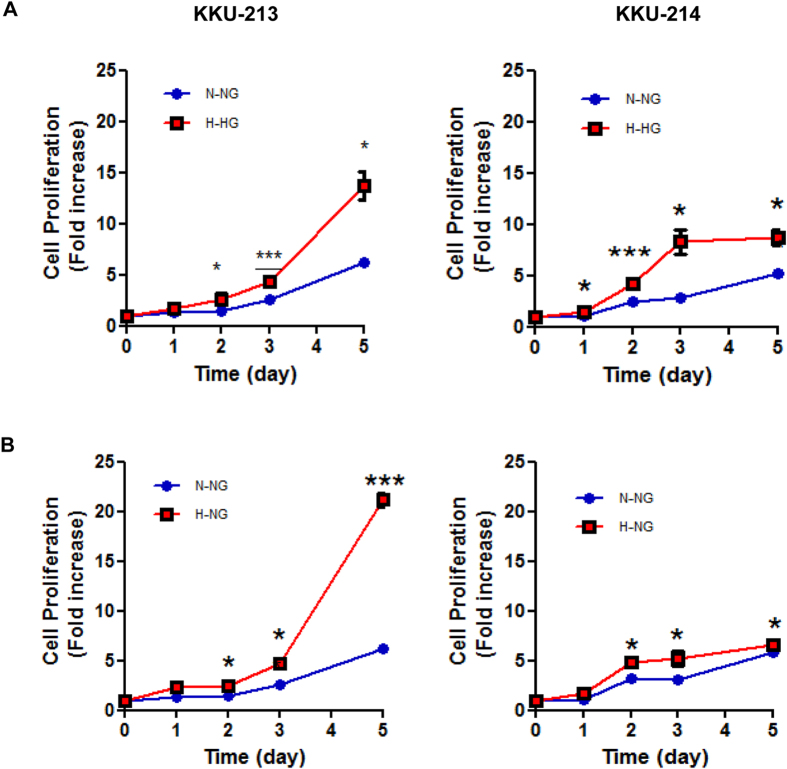
High glucose condition promotes proliferation of CCA cells. (**A**) HG cells cultured in high glucose media (H-HG) showed significantly higher proliferation rates than NG cells cultured in normal glucose media (N-NG). (**B**) Switching media by culturing NG cells in high glucose media (H-NG) significantly increased proliferation rates as compared to NG cells cultured in normal glucose media (N-NG). The data are mean ± SD of triplicate assays and is one representation of three independent experiments. NG and HG = cells continuously cultured in normal and high glucose media; **P < 0.01, ***P < 0.001.

NG cells and HG cells were cultured in the specified media for 5 days and cell numbers at days 1, 2, 3 and 5 were determined using the MTT assay. The proliferation rates of NG cells and HG cells were compared. As shown in Fig. 1A, the HG cells of both tested cell lines had significantly higher proliferation rates than the NG cells (*P* < 0.05). To assure that the growth enhancing effect was due to the glucose condition, the NG cells were switched to culture in the media with high glucose (H-NG). The proliferation rates of the H-NG cells were significantly increased compared to those of NG cells cultured in the normal glucose condition (N-NG) (*P* < 0.05; Fig. 1B).

### High glucose promotes adhesion, migration and invasion of CCA cells

**Figure 2:**
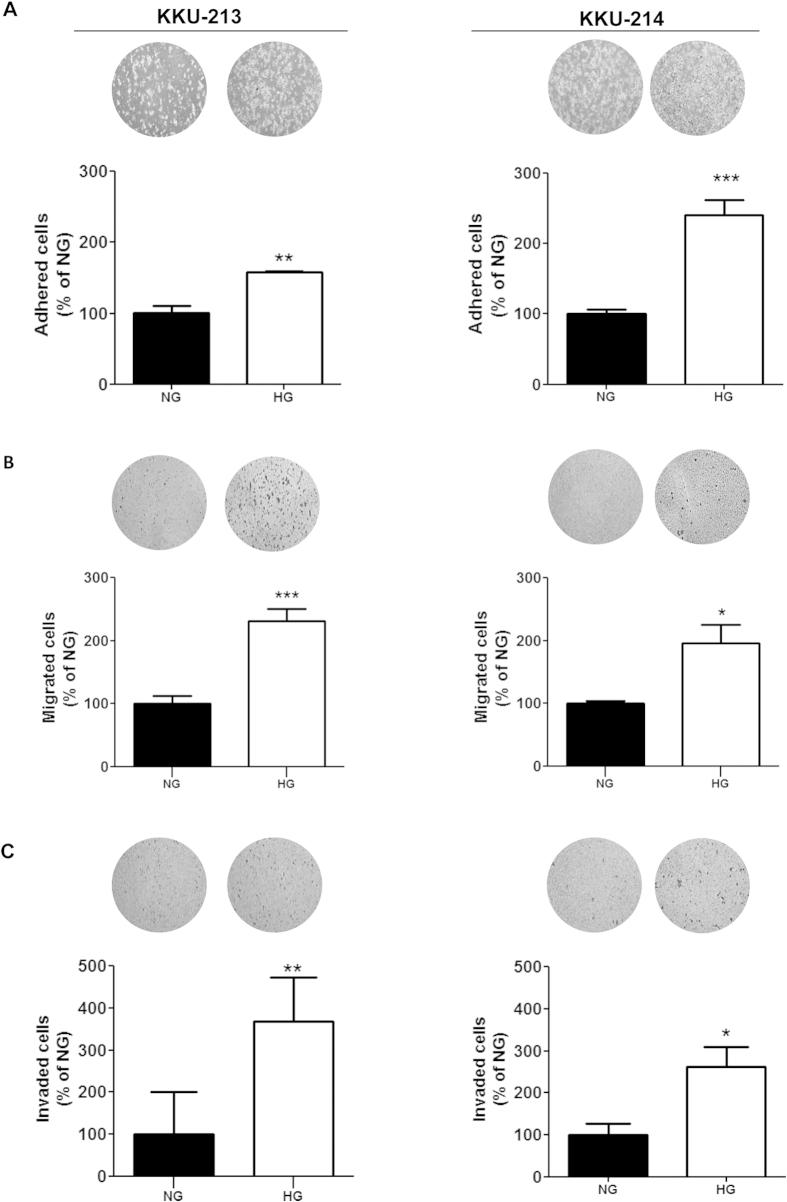
High glucose promotes metastatic related phenotypes of CCA cells. HG cells and NG cells were compared for (**A**) adhesion, (**B**) migration and (**C**) invasion. The data are mean ± SD of triplicates and is one representation of two independent experiments. NG and HG = cells continuously cultured in normal and high glucose media; *P < 0.05, **P < 0.01, ***P < 0.001.

Adherence to the extracellular matrix (ECM) is an important property for metastatic cells to localize at the secondary site, thus, the effect of high glucose on promoting cell adhesion to the ECM was next observed. CCA cells were allowed to adhere on a Matrigel pre-coated plate for 1 h and the numbers of adhered cells were determined. It is obvious that the number of adhered cells of the HG group were significantly higher than those of NG group (*P* < 0.05; Fig. 2A).

The effects of high glucose on cell motility and invasion were tested using the Boyden chamber assay. As shown in Fig. 2B, the migrated cells of HG cells from both KKU-213 and KKU-214 were almost double those of the NG cells (*P* < 0.05). Similar results were obtained for the invasion assay as the numbers of the invaded cells of the HG cells were significantly higher than those of NG cells (*P* < 0.05) (Fig. 2C).

### The phosphokinase array revealed STAT3 as a candidate pathway induced by high glucose

**Figure 3:**
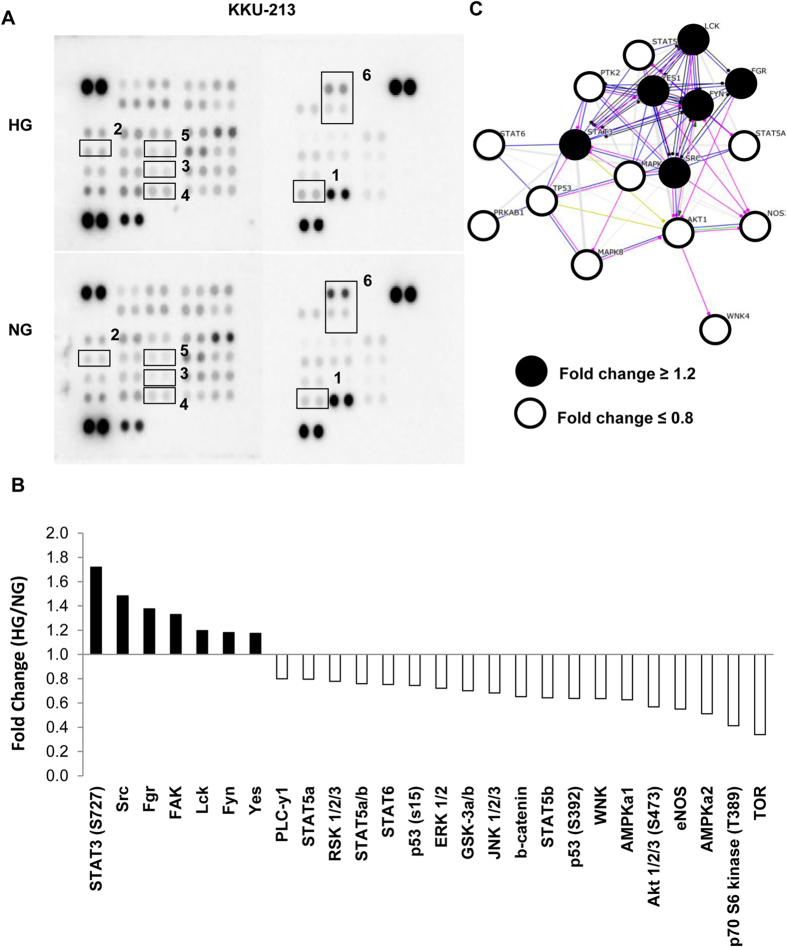
Differential phosphokinase profiles of HG cells vs. NG cells of KKU-213. The phosphokinase signaling (**A**) was screened in HG vs. NG cells of KKU-213 using a phospho-kinase array kit containing 46 phosphokinases. (**B**) The p-STAT3 (S727) is the dominant kinase induced by high glucose, followed by SFK and FAK. (**C**) The protein interaction prediction by String 9.1 suggests the interaction of STAT3 and SFK signaling pathways in HG cells. SFK = Src Family Kinases, FAK = focal adhesion kinase; 1 = STAT3 (S727), 2 = Src, 3 = Fgr, 4 = FAK, 5 = Lck, 6 = p53.

To examine the possible molecular signals that were activated by high glucose and related to progression of CCA cells, the phosphokinase array containing 46 specific antibodies to phosphokinases was used to screen the phosphorylation status of multiple cellular kinases. As KKU-213 showed a strong response to the high glucose condition, therefore cell lysates from NG and HG cells of KKU-213 were used for phosphokinase array screening. When HG to NG cells, were compared, there were 7 phosphokinases activated (signals >1.2 fold) and 19 phospho-kinases inactivated (signal <0.8) (Fig. 3A,B).

Among the phosphokinases activated, p-STAT3 (S727) was the highest activated signal in HG cells, followed by the Src Family Kinases (SFKs) (Src, Fgr, Yes, Lck) and the focal adhesion kinase (FAK). Contrarily, tumor suppressor proteins, *i.e*., p-p53 and p-AMPKα were inactivated in HG cells. The interactions of these kinases were then generated using String 9.1 (http://string-db.org, accessed on October 22, 2014) (Fig. 3C) and the REACTOME pathway was analyzed using the “Enrichment demand” of STRING 9.1. These results suggest the STAT3 pathway to be dominant in HG cells (Supplementary Table 1).

### High glucose promotes phosphorylation and nuclear localization of STAT3 in CCA cells

**Figure 4:**
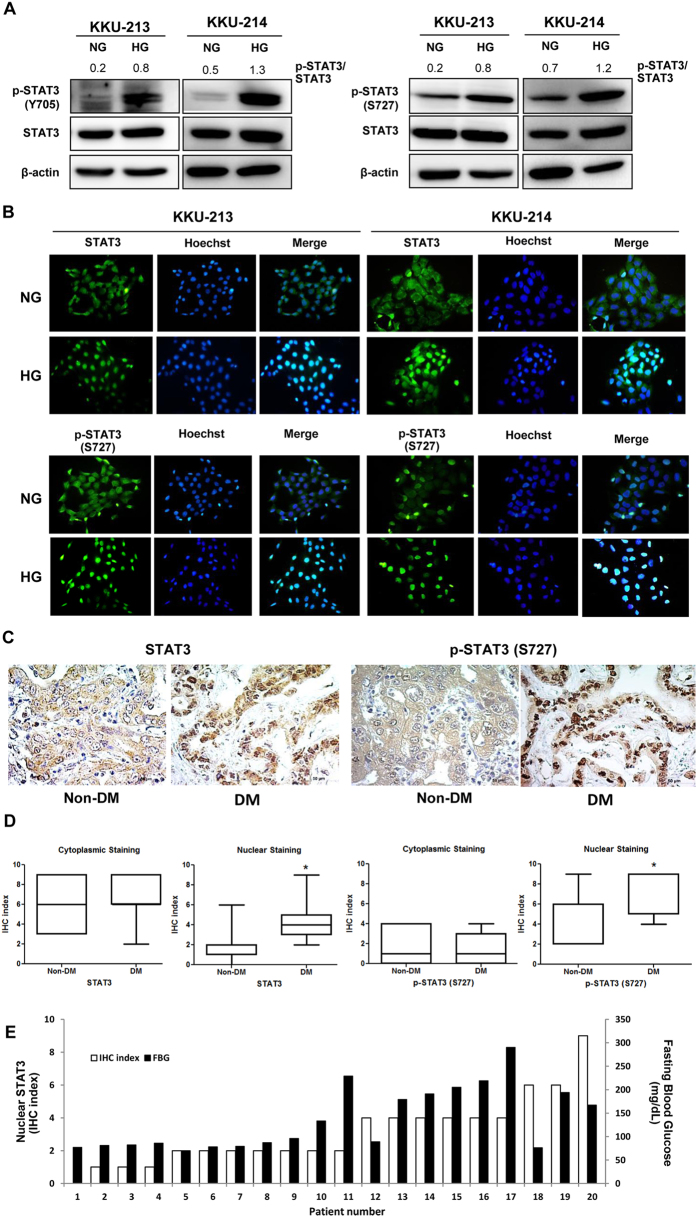
High glucose promotes phosphorylation and nuclear translocation of STAT3. Activation of STAT3 was verified in HG cells vs. NG cells of KKU-213 and KKU-214. (**A**) p-STAT3 of Y705 and S727 were determined using western blotting. β-Actin was used as an internal control and the numbers indicate the p-STAT3/STAT3 ratio. (**B**) Nuclear localization of STAT3 and p-STAT3 (S727) was demonstrated using immunocytofluorescent staining. Hoechst staining (blue) indicates nucleus; STAT3 and p-STAT3 conjugated FITC (green) indicate cytoplasmic or nuclear localization of STAT3 and p-STAT3; and the merged image (bright blue) indicates co-localization of STAT3 and p-STAT3 with Hoechst. (**C**) The immunohistochemistry of STAT3 and p-STAT3 in CCA patient tissues with DM (≥126 mg/dL; n = 9) and no DM (<126 mg/dL; n = 11) indicates significantly higher nuclear localization of STAT3 and p-STAT3 (S727) of tissues from CCA patients with DM than those without DM. (**D**) The quantification of immunohistochemistry of STAT3 and p-STAT3. (**E**) The immunohistochemistry index (IHC) of nuclear STAT3 in CCA tissues were correlated with the levels of fasting blood sugars of individual patients (Spearman’s Rho = 0.896, P < 0.01). NG and HG = cells continuously cultured in normal and high glucose media; *P < 0.05.

The activation of the STAT3 pathway in NG and HG cells of KKU-213 and KKU-214 was further verified by immunoblotting. As shown in Fig. 4A, the levels of p-STAT3 at both Y705 and S727 were significantly increased in HG cells while the levels of total STAT3 were not different. To ascertain the activation of STAT3 in HG cells, localization of STAT3 and p-STAT3 (S727) were determined using immunocytofluorescent staining. As shown in Fig. 4B, the HG cells of KKU-213 and KKU-214 exhibited nuclear localization of STAT3 and p-STAT3 (S727) whereas NG cells showed predominantly cytoplasmic localization.

### CCA tissues from patients with DM exhibited high activation of STAT3

To demonstrate the association of the high glucose condition and STAT3 activation in CCA patients, the immunohistochemistry of STAT3 and p-STAT3 (S727) were performed in tumor tissues from CCA patients with various histological types (Supplementary Table 2). Age and sex matched patients were divided into two groups according to the levels of preoperative fasting blood glucose (FBG)^14^ to be non-DM if FBG < 126 mg/dL (n = 11) and DM if FBG ≥ 126 mg/dL (n = 9). The ages (mean ± SD, range) of the non-DM (55 ± 20 years, 43–68 years) *vs*. DM groups (56 ± 19 years, 51–65 years) were not significantly different. The FBS levels (mean ± SD, range) of the non-DM group (83 ± 6 mg/dL, 70–96 mg/dL) *vs*. DM groups (200 ± 44 mg/dL, 133–290 mg/dL) were markedly different (*P *< 0.001). As shown in Fig. 4C,D, expression of nuclear STAT3 and nuclear p-STAT3 (S727) but not cytoplasmic STAT3 and cytoplasmic pSTAT3 (S727) of tumor tissues from CCA patients with DM were significantly higher than those with non-DM (P < 0.05). Regardless of the histological types of CCA, the association of blood glucose levels and STAT3 activation was tightly bound by the fact that the levels of nuclear STAT3 in tumor tissues were significantly correlated to the levels of patient blood glucose (Fig. 4E; Spearman’s Rho = 0.896, *P* < 0.01).

### Downstream target genes of STAT3 were up-regulated in the high glucose condition

**Figure 5:**
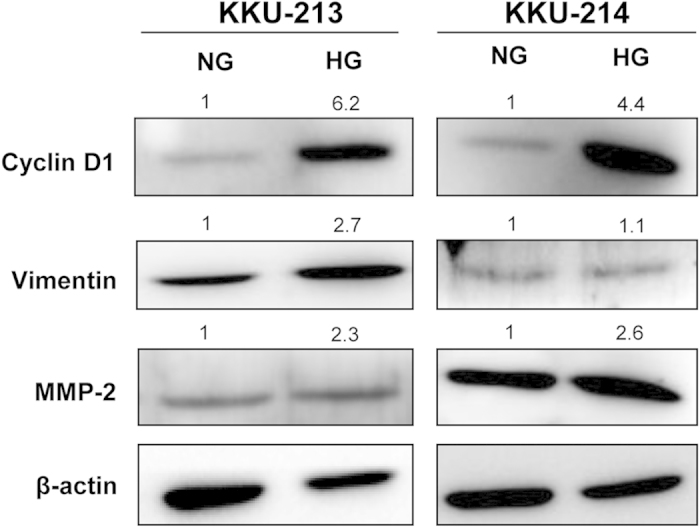
Downstream target genes of STAT3 related to proliferation, migration and invasion were up-regulated in HG conditions. Cyclin D1, vimentin and MMP2, the downstream targets of STAT3, were validated in NG vs. HG cells of KKU-213 and KKU-214 using western blotting. Quantification of each protein was performed using β-actin as an internal control. The expression of each protein was compared between NG and HG by giving NG = 1. The data one representation of two independent experiments. NG and HG = cells were continuously cultured in normal and high glucose media.

To confirm the association of STAT3 activation and the aggressive phenotypes observed in HG cells, the levels of p-STAT3 and its downstream target proteins related to proliferation (cyclin D1), migration (vimentin) and invasion (matrix metalloproteinase 2; MMP2) were determined. As shown in Fig. 5, all p-STAT3 downstream target proteins tested were up-regulated in the HG cells compared with those of the NG cells.

### Glucose when reduced to normal level decreases cell proliferation and STAT3 activation of CCA cells

**Figure 6:**
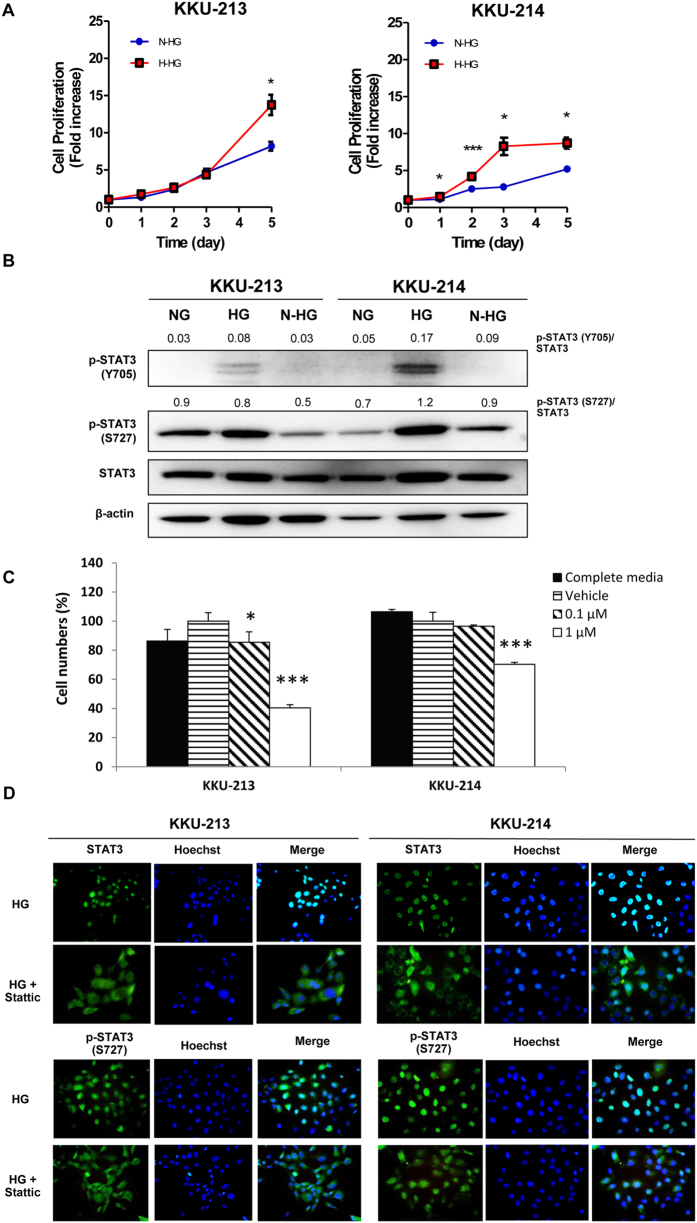
Controlling glucose level or using STAT3 inhibitor reduces the enhancing effect of high glucose on CCA cells. The HG cells cultured in the high glucose media (H-HG) were switched to be cultured in normal glucose media (N-HG) and compared for (**A**) cell proliferation; (**B**) expression of p-STAT3 (Y705 and S727). The numbers represent the pSTAT3 to STAT3 ratios of each band; (**C**) The treatment of Stattic (STAT3 inhibitor) significantly reduced proliferation and (**D**) inhibited nuclear translocation of STAT3 and p-STAT3 (S727) of HG cells. NG and HG = cells were continuously cultured in normal and high glucose media; *P < 0.05, ***P < 0.001.

It was further questioned as to whether glucose induced STAT3 activation can be controlled by the level of glucose. The HG cells were switched to culture in normal glucose media (N-HG). Cell proliferation and STAT3 activation of the N-HG cells were determined and compared with those of HG cells that were continued to be cultured in high glucose media (H-HG). The proliferation rate of N-HG cells was significantly reduced compared to those of H-HG (*P* < 0.05) (Fig. 6A). In addition, the levels of p-STAT3, both Y705 and S727, were significantly reduced to nearly the basal level of the NG cells when HG cells were switched to be cultured in a normal glĥucose condition (Fig. 6B).

### STAT3 inhibitor suppresses high glucose-induced growth in CCA cells

To verify whether a STAT3 inhibitor can minimize the effects of high glucose induced growth in CCA, Stattic, a well-known inhibitor for nuclear translocation of STAT3, was used. The HG cells of KKU-213 and KKU-214 were cultured in high glucose media in the presence of 0.1 and 1 μM of Stattic. Cells without Stattic and in the presence of vehicle were used as controls. As shown in Fig. 6C, the proliferation of CCA cells treated with Stattic was significantly suppressed (*P* < *0.05*) at 0.1 and 1 μM of Stattic for KKU-213 and 1 μM Stattic for KKU-214. The action of Stattic on inhibition of nuclear translocation of STAT3 and p-STAT3 (S727) was demonstrated by immunocytofluorescent staining (Fig. 6D).

## Discussion

In the present study, it is shown for first time that a high glucose condition could promote the proliferation and metastatic potential of CCA cells. The phosphokinase array data suggested that STAT3 activation was the dominant pathway responsible for the glucose enhancing progression of HG cells. The downstream targets of p-STAT3, *i.e*., cyclin D1, MMP2, and vimentin were increased in HG cells, supporting the roles of p-STAT3 in increasing cell proliferation, migration and invasion. The effect of high glucose on STAT3 activation was confirmed in tissues from CCA patients with DM who had a higher activation of STAT3 than that of non-DM patients. Finally, it was demonstrated that the effects of high glucose on progression of CCA cells could be reduced by either monitoring the levels of glucose or using a STAT3 inhibitor which subsequently reduced the aggression of CCA cells.

The evidence of diabetogenic conditions, i.e. high blood glucose, enhanced the risk and progression of several cancers has been increasingly demonstrated for a decade. The growing epidemiological evidence suggests DM as a risk factor for CCA development^10^,^11^,^15^, however, the effects of DM or a high glucose condition on CCA progression at the molecular level is limited.

High glucose levels are a new and neglected factor that links DM and cancer as an increased risk and progression of many cancers^16^,^17^. High glucose can promote many “hallmarks of cancer” phenotypes such as growth, metastatic potential and induction of specific isoforms of enzymes involving the predominant aerobic glycolysis or Warburg effect^18^. It is shown in the present study that CCA cells cultured in the high glucose condition had significantly higher rates of cell proliferation, adhesion, migration and invasion than those cultured in normal glucose. Moreover, switching cells cultured from normal glucose media to high glucose media enhanced cell proliferation. Similar observations were reported in several cancers, *e.g.,* cancers of breast, pancreas, prostate and colon^19^,^20^,^21^,^22^,^23^. It has been well recognized that the effects of glucose on cancer progression is an alternative function of glucose other than being the energy source^24^.

In the present study, comparisons of kinase signaling between CCA cells cultured in normal and high glucose conditions indicated that STAT3 activation was the dominant signaling pathway in HG cells. Phosphorylation of STAT3 at S727 and Y705 were highly activated in the cells cultured in high glucose as shown by the western blotting. The immunocytofluorescent staining of STAT3 and p-STAT3 (S727) affirmed this finding in such a way that HG cells exhibited significantly higher signals of nuclear STAT3 and p-STAT3 (S727) than the NG cells in both KKU-213 and KKU-214. The solid association of HG and STAT3 activation was strongly supported in CCA patients by the immunohistochemistry data showing that nuclear localization of STAT3 and p-STAT3 (S727) in CCA patient tissues with DM was significantly higher than those without DM. Moreover, the levels of STAT3 activation in tumor tissues were impressively correlated with the levels of blood glucose of CCA patients.

To link that the progressive phenotypes observed in HG cells were under STAT3 activation, the expressions of STAT3 downstream target proteins namely cyclin D1, vimentin and MMP2 were determined and compared between NG *vs*. HG CCA cells. The expression of these 3 proteins were representative of cell proliferation activation, migration and invasion, when they were significantly increased with high glucose. Phosphorylation of STAT3 upon high glucose activation was also reported in breast cancer cells^25^,^26^ and rat colon cancer cells^27^ resulting in increased cell proliferation and motility. Besides the activation of STAT3, JAK/STAT^26^,^27^, PI3K/AKT^29^ and wnt/β-catenin^25^, it was also reported to be promoted by high glucose as reported in cancers of breast, colon, pancreas and ovary. Therefore, the molecular mechanisms that responded to the activation of high glucose may be varied according to the type of cancer.

STAT3 is a transcription factor that plays important roles in the development and progression of many cancers. As a consequence, it becomes promising to be a therapeutic target of cancer treatment^28^,^29^. In CCA, STAT3 was reported to be a key molecule that gradually increased during the development of CCA as demonstrated in the liver fluke associated hamster model. High expressions of STAT3 and p-STAT3 (Y705) in CCA patient tissues were associated with poor prognosis. Patients with high STAT3 and p-STAT3 expression had shorter survival times than those with low expression of STAT3^30^. In addition, inhibition of STAT3 activation, significantly reduced the migratory activity of CCA cells^31^. These data suggested the association of STAT3 in the development and progression of CCA.

Apart from classical phosphorylation of Y705, the phosphorylation at S727 of STAT3 is needed for the maximal function in gene transcription^32^,^33^. The significant associations of p-STAT3 (S727) with tumorigenesis of prostate cancer and with the migration activity of sarcomatoid CCA^34^ have been emphasized^35^. The increased phosphorylation of p-STAT3 at Y705 and S727 in the HG cells as observed in the present study may imply the maximal activation of STAT3 in response to the high glucose condition.

**Figure 7:**
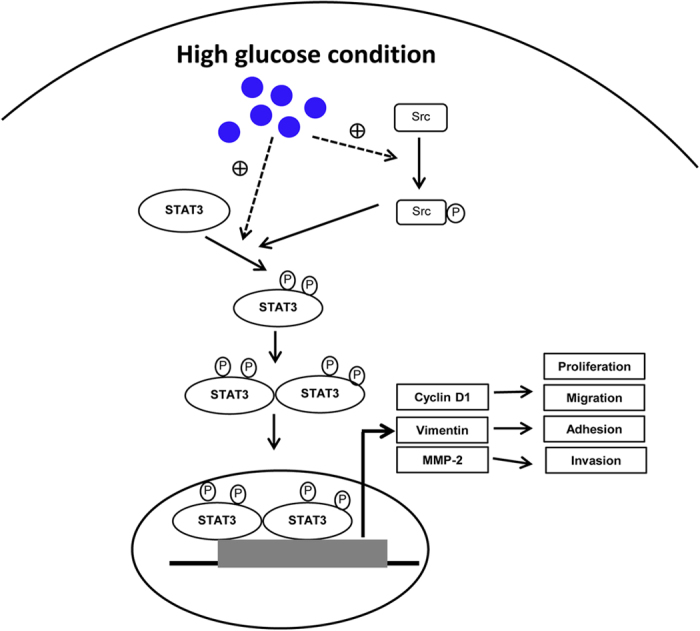
Molecular mechanism of high glucose promotes progression of CCA cells. High glucose levels promoted the phosphorylation and nuclear translocation of STAT3 which in turn activated the transcription of downstream target genes associated with aggressive phenotypes. High glucose also activated Src and stimulated STAT3 phosphorylation. Solid arrow = mechanism demonstrated in the present study; dashed arrow = unclarified mechanism.

Besides p-STAT3 (S727), up-regulation of p-Src (Y419) and p-SFKs were also activated under the HG condition. As Src is a kinase that can phosphorylate STAT3^36^, therefore increased STAT3 phosphorylation in HG cells may be, in part, stimulated by Src activation. Roles of Src and FAK in promoting proliferation and invasion were also reported in CCA^37^,^38^. The mechanism by which high glucose promotes progression of CCA cells via activation of STAT3 and Src is in turn the mechanism that activates the transcription of downstream target genes associated with aggressive phenotypes are summarized in Fig. 7.

The enhanced effect of high glucose on progression of CCA cells via STAT3 activation is demonstrated in the present study, both in cell lines and CCA patient tissues. Whether controlling the levels of glucose or reducing STAT3 activation could minimize the progressive phenotype, namely by inhibiting proliferation, was further explored. The proliferation rate and levels of p-STAT3 were markedly reduced to near the basal level when the HG cells were switched to culture in the normal glucose condition. The decreases of cell adhesion, migration and invasion were also expected when HG cells were cultured in normal glucose media. This expectation was based on the fact that 1) activation of STAT3 was tied to the level of glucose and 2) STAT3 regulated migration and invasion, thus inhibiting STAT3 activation using STAT3 inhibitors significantly decreased cell migration of CCA cell lines^39^.

Exposure to transient to excessively high levels of glucose could trigger the changes in epigenetic modification and induce long-lasting changes of gene expression in the experienced hyperglycemia cancer cells^40^. In addition, these “hyperglycemic memory” tumor cells exhibited significantly greater aggressive behavior (growth and metastasis) than those harvested from the control, both *in vitro* and in the mouse model^41^,^42^. These studies indicated that the effect of high glucose on cancer progression as stated in the present study and other reports is a consistent feature and the molecular mechanisms shown in cell lines and CCA patient tissues should be consistent *in vivo*.

In the current study, Stattic, a well-known inhibitor for nuclear translocation of STAT3 significantly reduced cell growth of HG cells cultured in the high glucose conditions. These data imply the possible clinical benefit of controlling blood glucose or STAT3 activation in CCA patients who have high blood glucose. The advantage of controlling blood glucose levels was shown in hepatocellular carcinoma (HCC) patients in such a way that the HCC patients with DM who could control blood glucose had significantly lower recurrence rates than those who could not^43^. From this perspective, either controlling blood glucose or using a STAT3 inhibitor may be a promising therapeutic strategy for CCA patients who have high blood glucose levels.

On the other hand, metformin, an antidiabetic drug, has been shown to exert the antitumor activity in many cancer types^44^,^45^ including CCA^46^. Metformin supplementation might then improve survival of CCA patients after operation^46^ and reduce risk of intrahepatic CCA among persons with DM^10^. A retrospective study of 250 DM patients who were newly diagnosed for CCA, however, showed that metformin did not improve the survival of these CCA patients^47^. Whether metformin might benefit CCA patients is an issue for future investigation.

In this study, there is also the limitation that the CCA cell lines and the vast majority of CCA cases presented in this study were associated with long-standing chronic inflammation from liver fluke (*Opisthorchis viverrini*) infection and hence, may not fully reflect CCAs with other risk factors. The effects of high glucose on progression of non-*Opisthorchis viverrini* related CCAs should be investigated.

In summary, this study highlights the enhancing effect of high glucose on progressive phenotypes of CCA cell lines. Activation of STAT3, increasing of p-STAT3 and nuclear translocation of p-STAT3, were shown to be some of the underlining mechanisms of the high glucose condition. These findings were demonstrated not only in CCA cell lines but also in CCA patient tissues. Controlling glucose levels or reducing STAT3 activation could minimize the enhancing effects of high glucose on progression of CCA, and may be of benefit in the treatment of CCA patients with high blood glucose.

## Materials and Methods

### Cell lines and CCA tissues

Human CCA cell lines; namely KKU-213 and KKU-214 were established from CCA patients and obtained from Japanese Collection of Research Bioresources (JCRB) Cell Bank, Osaka, Japan. All cell lines were cultured in Dulbecco’s Modified Eagle Medium (DMEM) (Gibco/Invitrogen, Calsbald, CA) with normal (N; 5.56 mM) or high (H; 25 mM) concentrations of glucose supplemented with 10% fetal bovine serum (Gibco/Invitrogen) and a 1% antibiotic-antimycotic (Gibco/Invitrogen). Cells were incubated in a 37 °C, 5% CO_2_, humidified incubator. All cell lines were cultured in N or H media for at least 5 passages allowing for their adaptation prior to use^48^. Cells harvested from the culture conditions for normal glucose media were designated as NG cells and those cultured in high glucose media were designated as HG cells.

Paraffin-embedded histologically proven CCA tissues (n = 20) were obtained from the specimen bank of the Liver Fluke and Cholangiocarcinoma Research Center, Faculty of Medicine, Khon Kaen University, Thailand. Written informed consent was obtained from each subject and the protocol has been reviewed and approved by The Khon Kaen University Ethics Committee for Human Research (HE571464) based on the Declaration of Helsinki and ICH-Good Clinical Practice Guidelines. The experiments were conducted in accordance with the approved protocols.

### Proliferation and Cytotoxic assay

Cell numbers were estimated using the MTT assay. Briefly, 3 × 10^3^ cells/well of NG or HG cells were seeded in a 96-well plate containing normal or high glucose media and incubated for 12 h. Then the media was replaced with specified media conditions and incubated further for 1, 2, 3 and 5 days with media changing at day 3 to prevent the effect of insufficient nutrients. At the completed incubation time, media was removed and 110 μL of 0.5 mg/mL MTT (Invitrogen, Carlsbad, CA) in PBS was added. Cells were further incubated for 4 h at 37 °C, 5% CO_2_. The formazan crystals were dissolved in dimethylsulfoxide and the optical density was measured at 540 nm using a microplate reader (SUNRISE, Groedig, Austria).

A STAT3 inhibitor, Stattic (Santa Cruz Biotechnology, Santa Cruz, CA) was used to inhibit the activation and nuclear localization of STAT3^49^. Cells in high glucose media were seeded into a 96-well plate and incubated overnight. Then cells were changed into the media containing 0.1 or 1 μM of Stattic and incubated for a further 72 h. The effect of Stattic on cell proliferation was examined using the MTT assay.

### Adhesion assay

A 96 well-plate was pre-coated with 100 μL of 50 μg/mL of Matrigel (BD Biosciences, Bedford, MA) at 37 °C, 5% CO_2_, overnight. After blocking with 3% bovine serum albumin in PBS for 1 h, cells (2 × 10^4^ cells/well) in serum free media were seeded and allowed to adhere for 1 h at 37 °C, 5% CO_2_. The adhered cells were fixed with 4% paraformaldehyde and stained with 0.5% crystal violet. The stain was solubilized with 2% sodium dodecylsulfate in distilled water and the absorbance was measured at OD540.

### Migration and invasion assay

CCA cell lines, KKU-213 and KKU-214 (30,000 cells/well) in serum free media were seeded in the upper Transwell^®^chambers (Corning, Lowell, MA) with complete media as a chemoattractant in the lower chamber. The plates were incubated at 37 °C, 5% CO_2_; 6 h for KKU-213 and 17 h for KKU-214. The migrated cells were fixed with 4% formaldehyde and stained with 0.1% sulforhodamine B. The migrated cells were photographed and counted under a low power microscope using Image J software (National Institutes of Health, USA).

The invasion assay was performed in the same manner as the migration assay except the upper chamber was pre-coated with 50 μg/well of Matrigel overnight before seeding cells. The invaded cells were determined after 6 h incubation for KKU-213, and 23 h for KKU-214.

### Phospho-kinase array

The human phospho-kinase array kit (ARY003B, R&D system, Minneapolis, MN) containing 46 phosphokinases printed in duplication was used to screen the signaling pathway. Briefly, the 80% confluent NG or HG cells were lysed with NP-40 lysis buffer containing phosphatase- and protease-cocktail inhibitors (Roche, Mannheim, Germany) and incubated at 4 °C for 15 min. Cell lysate was obtained after centrifugation at 12,000 xg, 4 °C, for 15 min and the total protein was quantified using Bio-Rad Protein Assay Dye Reagent Concentrate (Bio-Rad, Hercules, CA) according to the instructions of the manufacturer. Total protein lysate (600 μg) was incubated with an antibody-array membrane at 4 °C, overnight. The membrane was then incubated with cocktail-detection antibody and streptavidin horseradish peroxidase. The signals were detected by Chemireagent provided in the same kit and quantified using a Image Quant™ Imager (GE Healthcare Bioscience AB, Uppsala, Sweden).

### Immuno-blot analysis

The antibodies used in this study were anti-vimentin (ab137231) (Abcam, Cambridge, UK), anti-β-actin (A5441) (Sigma, St. Louis City, MO), anti-STAT3 (C-20), anti-p-STAT3 (Y705) (B-7), anti-p-STAT3 (S727) (S727-R), anti-Cyclin D1 (H-295) and anti-MMP2 (H-79) (Santa Cruz Biotechnology).

Cell lysate was collected and quantified for protein concentration as mentioned above. Total protein of 30 μg was subjected to a 10% SDS-polyacrylamide gel electrophoresis and transferred to a Hybond™-P PVDF membrane (GE Healthcare, Buckinghamshire, UK). The membrane was probed with each primary antibody at 4 °C overnight and HRP-conjugated secondary antibody (GE Healthcare) for 1 h at room temperature. The signals were detected using an enhanced chemiluminescence prime Western blotting detection kit (GE Healthcare). Image analysis was performed using the Image Quant™ Imager (GE Healthcare).

### Immunocytofluorescent staining

CCA cells were seeded in a Matrigel pre-coated slide chamber with a density of 1 × 10^4^ cells/well. Cells were seeded and incubated at 37 °C, 5% CO_2_ for 72 h. Cells were then fixed with 4% paraformaldehyde and permeabilized with 0.2% Triton X-100. Nonspecific antigens were blocked with 5% fetal bovine serum. The fixed cells were incubated with 1: 100 anti-STAT3 or anti-p-STAT3 (S727) antibodies at 4 °C overnight and FITC-conjugated secondary antibody (1: 200) (Santa Cruz Biotechnology) and 1: 10,000 Hoechst (Invitrogen, Eugene, OR), at room temperature for a further 2 h. The fluorescent imaging was obtained using a fluorescence microscope (ECLIPSE Ni-U; Nikon, Tokyo, Japan) with Nikon NIS-Elements software.

For the effect of Stattic on localization of STAT3 and p-STAT3 (S727), seeded cells were treated with 1 μM of Stattic (Santa Cruz Biotechnology) for 48 h before subjecting to the immunocytofluorescent staining.

### Immunohistochemistry

STAT3 and p-STAT3 (S727) were detected in the paraffin-embedded CCA tissues using a standard immunohistochemistry protocol. Briefly, the tissues were incubated with 1: 100 of anti-STAT3 or anti-p-STAT3 (S727) at room temperature, overnight. The Envision-Plus HRP system (Dako, Carpinteria, CA) against the primary antibody was applied at room temperature for 2 h. The peroxidase activity was observed using diaminobenzidine tetrahydroxychloride solution (DAB; Dako) as a substrate, and counter stained with hematoxylin. The frequency of target molecules were semi-quantitatively scored on the basis of percentage of the positively stained cells as 0 = negative; 1–25% = 1; 26–50% = 2; and >50% = 3. The intensity of stained cells was scored as weak = 1, moderate = 2 and strong = 3. The immunohistochemistry (IHC) index was calculated as frequency × intensity.

### Statistical analysis

All quantitative data are shown as mean ± SD of a representative from at least two independent experiments. Triplicate tests were performed for each experiment. The two-tail Student’s *t* test was used to compare the differences between two groups. *P *< 0.05 was considered as statistical significance.

## Additional Information

**How to cite this article**: Saengboonmee, C. *et al.* High glucose enhances progression of cholangiocarcinoma cells *via* STAT3 activation. *Sci. Rep.* **6**, 18995; doi: 10.1038/srep18995 (2016).

## Supplementary Material

Supplementary Tables 1-2
