# Supplementary material for: High glucose enhances progression of cholangiocarcinoma cells via STAT3 activation
Source: Sci Rep. 2016 Jan 8;6:18995. doi: 10.1038/srep18995 (PMC4705543; doi:10.1038/srep18995)
Supplement: Supplementary Tables 1-2 [file srep18995-s1.pdf]

High glucose enhances progression of cholangiocarcinoma cells *via* STAT3 activation

Running title: High glucose enhances progression of cholangiocarcinoma

Charupong Saengboonmee, BSc[1,4], Wunchana Seubwai, PhD[2,4], Chawalit Pairojkul, MD [3,4], Sopit Wongkham, PhD[1,4*]

Supplementary Table 1 Suggested pathways for HG cells using REACTOME analysis

Term	**p-value**	**n**
Dissociation and translocation of STATs to the nucleus	$1.56e^{-13}$	6
p-JAK: SFKs: p-KIT complex: p-STAT dimers	$1.56e^{-13}$	6
p-JAK: SFKs: p-KIT complex: p-STATs	$1.56e^{-13}$	6
Phosphorylation of STATs	$3.89e^{-13}$	6
p-JAK: SFKs: p-KIT complex: STATs	$3.89e^{-13}$	6
Signaling by SCF-KIT	$2.46e^{-9}$	6
CTLA4 inhibitory signaling	$4.93e^{-7}$	4
PECAM1 interaction	$8.21e^{-7}$	4

Supplementary Table 2 Demographic information of CCA patients with DM and non-DM

**Variables**		**Non-DM**	**DM**
Age (Year)	< 56	5	4
	≥ 56	6	5
Sex	Female	2	4
	Male	9	5
Histopathology	Well differentiated	7	5
	Moderately differentiated	4	3
	Poorly differentiated	0	1
TNM stage	III	9	7
	IV	2	2
Metastasis	Absence	9	8
	Presence	2	1
